# High-Flow Nasal Cannula Oxygenation in Older Patients with SARS-CoV-2-Related Acute Respiratory Failure

**DOI:** 10.3390/jcm10163515

**Published:** 2021-08-10

**Authors:** Arthur Hacquin, Marie Perret, Patrick Manckoundia, Philippe Bonniaud, Guillaume Beltramo, Marjolaine Georges, Alain Putot

**Affiliations:** 1 Department of Geriatric Internal Medicine, Dijon University Hospital, 21000 Dijon, France; arthur.hacquin@chu-dijon.fr (A.H.); marie.perret@chu-dijon.fr (M.P.); patrick.manckoundia@chu-dijon.fr (P.M.); 2 Department of Pulmonary Medicine and Intensive Care Unit, University Hospital, 21000 Dijon, France; philippe.bonniaud@chu-dijon.fr (P.B.); guillaume.beltramo@chu-dijon.fr (G.B.); marjolaine.georges@chu-dijon.fr (M.G.)

**Keywords:** COVID-19, pneumonia, coronavirus, comfort, mortality

## Abstract

We aimed to compare the mortality and comfort associated with high-flow nasal cannula oxygenation (HFNCO) and high-concentration mask (HCM) in older SARS-CoV-2 infected patients who were hospitalized in non-intensive care units. In this retrospective cohort study, we included all consecutive patients aged 75 years and older who were hospitalized for acute respiratory failure (ARF) in either an acute geriatric unit or an acute pulmonary care unit, and tested positive for SARS-CoV-2. We compared the in-hospital prognosis between patients treated with HFNCO and patients treated with HCM. To account for confounders, we created a propensity score for HFNCO, and stabilizing inverse probability of treatment weighting (SIPTW) was applied. From March 2020 to January 2021, 67 patients (median age 87 years, 41 men) were hospitalized with SARS-CoV-2-related ARF, of whom 41 (61%) received HFNCO and 26 (39%) did not. Age and comorbidities did not significantly differ in the two groups, whereas clinical presentation was more severe in the HFNCO group (NEW2 score: 8 (5–11) vs. 7 (5–8), *p* = 0.02, and Sp02/Fi02: 88 (98–120) vs. 117 (114–148), *p* = 0.03). Seven (17%) vs. two (5%) patients survived at 30 days in the HFNCO and HCM group, respectively. Overall, after SIPTW, HFNCO was significantly associated with greater survival (adjusted hazard ratio (AHR) 0.57, 95% CI 0.33–0.99; *p* = 0.04). HFNCO use was associated with a lower need for morphine (AHR 0.39, 95% CI 0.21–0.71; *p* = 0.005), but not for midazolam (AHR 0.66, 95% CI 0.37–1.19; *p* = 0.17). In conclusion, HFNCO use in non-intensive care units may reduce mortality and discomfort in older inpatients with SARS-CoV-2-related ARF.

## 1. Introduction

SARS-CoV-2 has infected millions of individuals worldwide, but its burden has been particularly heavy in the older population. Nearly one-third of older patients hospitalized with SARS-CoV-2 pneumonia die in hospital [1,2]. Most deaths are the result of acute respiratory failure (ARF) linked to viral pneumonia, for which optimal therapeutic management is still a matter of debate. Many of these patients are admitted to intensive care units (ICUs) because they require mechanical ventilation. Older comorbid patients are disproportionately affected and at a much higher risk of death. However, they are frequently refused ICU access, especially in the current context of scarce resources [3]. Older patients with ARF are thus frequently hospitalized outside the ICU, requiring alternatives to tracheal intubation. In this context, aside from drug therapies, high-flow nasal cannula oxygenation (HFNCO) was suggested as a promising non-invasive tool for SARS-CoV-2-related ARF [4].

HFNCO, delivering up to 60 L/min of oxygen, is a well-documented device in the supportive care of hospitalized patients with ARF, improving pre-oxygenation when intubation is needed and reducing mortality [5,6]. However, side effects have been reported (nasal bridge ulceration, pneumothorax, epistaxis) [7], though they have only been partially assessed in frail older adults. These side effects are comparable with those under conventional oxygen devices: mask discomfort, nasal, and oral dryness, eye irritation, nasal and eye trauma, bronchoconstriction, and gastric distention [8]. Few studies have focused on the impact of HFNCO in older patients [9,10,11], and, to the best of our knowledge, only one of them studied the impact of HFNCO during SARS-CoV-2 ARF [10]. However, this study was observational with no comparison group.

The absence of evidence for SARS-CoV-2 ARF management in frail older populations and the disparities in available care resources worldwide have led to a disconcerting heterogeneity of practices. There is an urgent need for specific data in older patients with this life-threatening and now frequent condition.

Since March 2020, older patients admitted to our hospital in the acute geriatric unit (AGU) or the acute pulmonary care unit (APCU) for SARS-CoV-2-related ARF have been treated either with a high-concentration mask (HCM) or with HFNCO, depending on their oxygen needs and symptoms, as well as equipment availability.

In this study, we aimed to investigate the impact of HFNCO compared to high-concentration mask (HCM) oxygen therapy on the survival and comfort of patients hospitalized for SARS-CoV-2-related ARF outside the ICU.

## 2. Materials and Methods

### 2.1. Study Design

We performed an observational retrospective study using hospital records from the AGU and APCU of a French 1800-bed University Hospital. Patients were admitted during the first two waves of the pandemic from March 2020 to January 2021. Participants (*n* = 67) were categorized into two groups, either receiving HFNCO (*n* = 41) or receiving HCM (*n* = 26). In-hospital 30-day survival was the primary outcome.

To investigate the comfort of patients undergoing HFNCO, we considered several secondary outcomes. Morphine is used to relieve the symptoms of dyspnea and enhance comfort in ARF [12]; therefore, we aimed to evaluate the impact on dyspnea by comparing the morphine prescription between the two groups.

Midazolam is used in our units for sedation of terminally ill patients [13], as stated in French Law, but also and more frequently, at lower titration, to relieve the symptoms of anxiety [14], which are especially frequent in ARF patients. The association of HFNCO with anxiety and restlessness was thus evaluated by comparing the prescription of midazolam between the two groups.

### 2.2. Patients

We included all consecutive patients aged 75 years or older hospitalized for SARS-CoV-2 ARF in the AGU or the APCU of our hospital.

ARF was defined as having a respiratory rate superior to 30 breaths per minute, labored or paradoxical breathing, signs of hypercapnia, or difficulty talking [15]. SARS-CoV-2 infection confirmed by a positive real-time polymerase chain reaction (RT-PCR) was mandatory. Patients were excluded if invasive ventilation or exclusive palliative care were decided before HFNCO/HMC or if there was a contraindication to HFNCO (consciousness disorder, claustrophobia, upper airway obstruction, facial trauma or deformity, abundant sputum, or emesis) [16]. Patients who were admitted to the ICU during their hospital stay were also not included.

### 2.3. Data Collection

The data were collected through a manual review of each participant’s medical record. Demographic data, site of SARS-CoV-2 pneumonia acquisition (i.e., community acquired or nursing-home acquired pneumonia), medical history, clinical, biological features, and acute treatment at baseline (i.e., ARF onset) were extracted from electronic or handwritten medical records. The Charlson comorbidity index was computed retrospectively using patient medical history [17]. The NEW2 prognostic score [18], which was developed to predict in-hospital mortality using 5 clinical variables (respiration rate, oxygen saturation, systolic blood pressure, pulse rate, and level of consciousness or new confusion) and the WHO severity scale [19], specifically dedicated to COVID-19 (S1: no pneumonia; S2: pneumonia, with SpO2 ≥ 90% on room air; S3: severe pneumonia, with respiratory rate > 30 breaths/min or SpO2 < 90% on room air; and S4: critical disease, with acute respiratory distress syndrome) were also calculated. To appreciate hypoxemia, we used the SpO2/FiO2 ratio, which has been broadly used during the COVID-19 pandemic [20], and collected respiratory rate, heart rate, and temperature. The degree of radiological damage (in percentage) on thoracic CT was obtained from standardized radiologist reports. For analysis purposes, the WHO severity scale and radiological damage on thoracic CT scan were computed as binary covariates, respectively, <S3 or more and <50% of radiological damage or more.

This study was strictly retrospective and observational, and as such, it did not require approval by an Institutional Review Board, in accordance with Article L1121-1 of the French Public Health Code (Law n°2012-300, dated 5 March 2012).

### 2.4. Statistical Analysis

#### 2.4.1. Missing Values

A manual review was performed for all missing data. There were no missing data for the main outcome variables: comorbidity, RT-PCR date, admission date, or first administration of HMC and HFNCO. We considered missing values as missing at random, and 10 datasets were imputed using multiple imputation with the predictive mean matching method [21].

#### 2.4.2. Description of Covariates

Continuous variables are described as medians (interquartile range (IQR)) for the continuous variables and the numbers (percentage) for categorical variables. The Wilcoxon rank test, $\chi^{2}$ or Fisher’s exact test and the log-rank test were computed to compare continuous, dichotomized, and survival variables, respectively.

#### 2.4.3. Propensity Score

Older patients who are put on HFNCO often have a more severe presentation than patients with HCM. To account for potential confounders relative to the association of HFNCO use and mortality, we created a propensity score for each patient and applied stabilized inverse probability weighting (SIPW) [22]. To do so, we first computed a non-parsimonious logistic regression where HFNCO was the explanatory variable. The covariates were selected based on their influence on HFNCO according to the literature and the differences in baseline characteristics [23]. The balance between the HCM and HFNCO groups were verified graphically for covariates.

To adjust for confounders, we computed SIPW-adjusted Kaplan–Meier curves for in-hospital 30-day survival, and morphine and midazolam prescription. The Cox proportional hazard regression was then weighted on the SIPW to construct a marginal structural Cox model [24]. Statistical tests were two-tailed, and a *p*-value of <0.05 was considered significant.

The data management and statistical analyses were performed using Rstudio version 1.3.1073 (RStudio Team, PBC, Boston, MA, USA).

## 3. Results

### 3.1. Population

A total of 67 consecutive patients aged ≥75 years were included (37 in the AGU group and 30 in the APCU group). These patients were admitted to hospital for SARS-CoV-2-related ARF between 3 March 2020 and 27 January 2021.

The baseline characteristics are presented in Table 1. The median age was 86 years (range of 77–99 years; (IQR) 84–89 years). There was no significant difference in age between patients in the HCM and HFNCO groups.

The type of hospital unit differed significantly between the two groups (27 patients (66%) received HFNCO in the APCU vs. 14 (34%) in the AGU *p* < 0.001). When compared to AGU patients, APCU patients were more frequently men (23 (77%) vs. 18 patients (46%), *p* = 0.037) and lived less frequently in nursing homes (5 (17%) vs. 19 patients (51%), *p* = 0.025). The corticosteroids were more often administered at baseline in the APCU (29 patients (96%) vs. 23 (64%) *p* = 0.003) and there was a trend toward better in-hospital survival (seven patients (33%) in APCU vs. two patients (5%) in AGU, *p* = 0.08).

A comparison of HCM and HFNCO patients showed that there was no significant difference in comorbidities except for neurocognitive disorders, which were less frequent in the HFNCO group (5 patients (12%) vs. 14 patients (54%) in the HCM group, *p* < 0.001).

Although the median NEW2 score was lower in the HFNCO group (8 (5–11) vs. 7 (5–8) in the HCM group *p* = 0.016), the WHO severity score did not differ significantly between the groups: 34 patients (83%) in the HFNCO group had a WHO severity score ≥S3 vs. 14 (76%) patients in the HCM group, *p* = 0.7.

The baseline respiratory rate was elevated in both groups (26 (21–35) per minute vs. 31 (24–36) per minute, *p* = 0.3). However, the median SpO2/FiO2 was lower in the HFNCO group (88 (98–120) vs. 117 (114–148) in the HCM group, *p* = 0.03).

**Figure 1:**
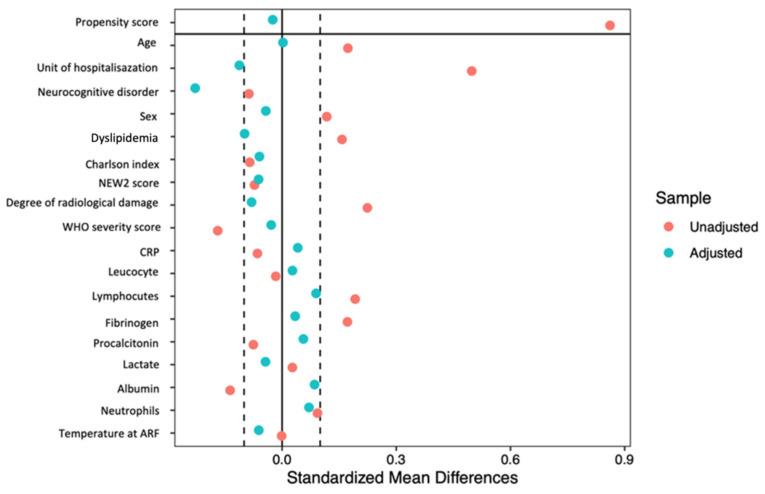
Unadjusted and stabilized inverse probability weighting-adjusted standard mean difference of covariates at baseline.

After SIPTW, the differences in the baseline covariates that were apparent in the overall sample (including unit of hospitalization) were no longer present (Figure 1).

### 3.2. Outcomes

Overall, seven (17%) patients in the HFNCO were alive at 30 days vs. only two (5%) patients in the HCM group. The in-hospital mortality was related to COVID-19 respiratory failure in all cases. During the hospital stay, 49 (73%) patients received morphine (26 (68%) in the HFNCO group vs. 23 (92%) in the HCM group), with a median time before introduction of 4 (1–31) days, and 46 (69%) received midazolam (26 (67%) in the HFNCO group vs. 20 (80%) in the HCM group), with a median time to introduction of 6 (2–31) days.

The associations between HFNCO and the main and secondary outcomes, before and after SIPW, are presented in Table 2. The in-hospital 30-day survival (HR 0.57 (95% CI 0.33–0.99), *p* = 0.04) and time to morphine introduction (HR 0.39 (95% CI 0.21–0.71), *p* = 0.002) were significantly associated with HFNCO after SIPW. The time to midazolam introduction (HR 0.66 (95% CI 0.37–1.19), *p* = 0.17) did not significantly differ between the two groups. After stratification by hospital unit, the weighted HRs for in-hospital mortality were similar in the AGU (HR 0.62 (95% CI 0.25–1.10), *p* = 0.09) and APCU (HR 0.43 (95% CI 0.13–1.37), *p* = 0.15).

**Figure 2:**
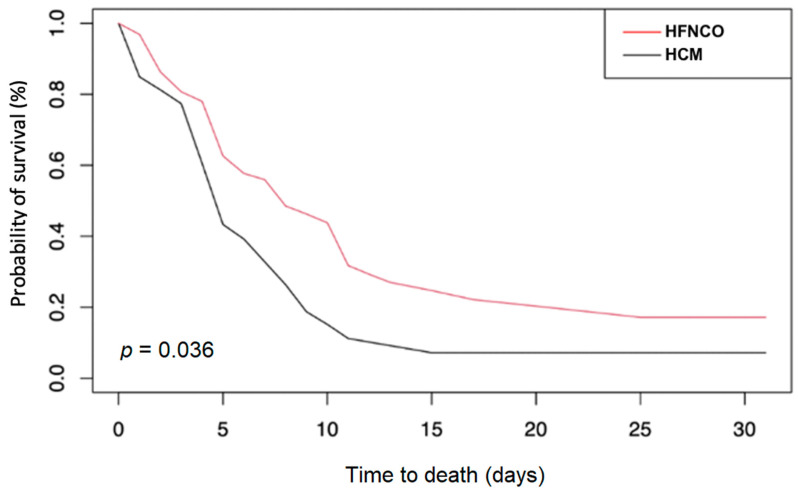
SIPW-adjusted Kaplan–Meier curves of 30 day mortality in older patients with high-flow nasal cannula oxygenation (HFNCO) versus high-concentration mask (HCM).

The SIPW-adjusted Kaplan–Meier curve for in-hospital survival is presented in Figure 2. Although a majority of patients died within 10 days in both groups, HFNCO was associated with higher 30-day survival (*p* = 0.036).

## 4. Discussion

In this study of older patients hospitalized outside the ICU for SARS-CoV-2-related ARF, only 13% survived at 30 days, with most of the deaths occurring within the 10 days following the first positive SARS-CoV-2 RT-PCR result. In the majority of patients, respiratory discomfort during the hospital stay required morphine introduction, and midazolam was administered for sedation/anxiolysis. Our observational data suggest that HFNCO may improve the prognosis as mortality was reduced by nearly half.

Although several authors have already described the poor prognosis of older patients with SARS-CoV-2 ARF [2,25], to the best of our knowledge, this is the first study focusing on the use of HFNCO in older adults. These findings are of particular importance for patients refused ICU admission, an ethically problematic situation that occurred frequently during the pandemic due to ICU congestion following the extreme variations in incidence rates [3].

In situations with such a poor prognosis, acute care should focus at least as much on patient quality of life as on survival. Therefore, we aimed not only to assess whether HFNCO improved mortality but also if it was associated with reduced respiratory discomfort. Of note, mortality was related to COVID-19 respiratory failure in all cases. No side effects of oxygen device were reported in either group. Our results suggest that HFNCO tends to reduce the morphine requirement, a surrogate marker of discomfort in this study. Several studies evaluating the impact of HFNCO on comfort [26,27,28], comparing dryness of the ENT area [26] or visual analogic scale ratings [27], obtained contradictory results, and none of them focused on older patients. To the best of our knowledge, this study is also the first to investigate the impact of HFNCO on COVID-19-infected patients.

Midazolam is frequently used for anxiety or sedation purposes in older patients with ARF; however, its use did not significantly differ between the HFNCO and HCM groups. The high level of technical care, lack of family presence and isolation, and fear of death in those conditions lead to extreme levels of anxiety in our patients [29]. Anxiety cannot be curbed using ventilation alone, and treatments such as midazolam are often required. Moreover, midazolam is used at higher doses for another purpose in our units, namely sedation until death in terminally ill patients in cases of care-resistant distress [30], as specified by French Law.

Most of the reports concerning SARS-CoV-2 ARF are obtained from ICUs, but older patients were often denied access to such settings. One of the strengths of this study is the use of non-ICU data, so these results can be extrapolated to most medical units admitting older patients with severe COVID-19.

We acknowledge that there are some limitations to our work. Although this study included all consecutive patients suffering from SARS-CoV-2-related ARF, we were able to include only 67 patients aged ≥75 years. Nevertheless, this limited number of patients was sufficient to highlight a significant difference in both mortality and morphine consumption. Second, the patients tended to have a more severe presentation in the APCU than in the AGU. However, the propensity score method enabled us to take into account the main confounders, including neurocognitive disorders and hospitalization unit, the two main features that differed between the groups. Despite an adjustment for confounders, we cannot exclude that patients from the two groups had different prognoses at the baseline, given the observational design. However, an observational and pragmatic approach was preferred to a randomized trial, which would have raised serious feasibility and ethical issues. Given the retrospective design and the limited size, these preliminary results need to be confirmed.

## 5. Conclusions

Ensuring patient comfort and weighing the benefit–risk balance are major parts of a clinician’s work when faced with SARS-CoV-2-related ARF in older patients who are not able to access an ICU. High-flow nasal cannula oxygenation can help decrease mortality in older patients suffering from this dramatic clinical situation, while reducing discomfort with a lower morphine requirement. Further studies need to be performed on a larger scale to confirm these results.

## Figures and Tables

**Table 1:** Baseline characteristics (*n* (%) or median (interquartile range), unless stated otherwise).

		HCM *n* = 26	HFNCO *n* = 41	*p*
**Demographics**				
Acute geriatric unit		23 (88)	14 (34)	<0.001
Acute pulmonary care unit		3 (12)	27 (66)	
Age (years)		86 (84–89)	87 (85–90)	0.785
Men		14 (54)	27 (66)	0.468
CAP		13 (50)	31(76)	0.172
NHAP		13 (50)	10 (24)	
**Medical history**				
High blood pressure		20 (76.9)	31 (75.6)	1.000
Dyslipidemia		7 (26.9)	14 (34.1)	0.726
Diabetes		11 (42.3)	14 (34.1)	0.679
Atrial fibrillation		10 (38.5)	11 (26.9)	0.465
Neurocognitive disorder		14 (53.8)	5 (12.2)	0.001
Chronic kidney disease		8 (30.8)	11 (26.8)	0.944
Chronic respiratory failure		3 (11.5)	3 (7.3)	0.880
Charlson index (mean (SD))		2.81 (2.58)	2.17 (2.01)	0.262
**Degree of radiological damage**				
	<10%	1 (1.5)	5 (7.4)	0.884
	10–25%	4 (5.9)	7 (10.4)	
	25–50%	9 (13.4)	12 (17.9)	
	50–75%	4 (5.9)	9 (13.4)	
	>75%	2 (2.9)	3 (4.4)	
**Severity scores**				
WHO severity score S1		1 (1.5)	3 (4.4)	0.650
WHO severity score S2		5 (7.4)	4 (5.9)	
WHO severity score S3		15 (22.4)	24 (35.8)	
WHO severity score S4		6 (8.9)	9 (13.4)	
NEW2 score		8 (5.25–11)	7 (5–8)	0.016
**Clinical examination**				
Respiratory rate (bpm)		31(24–35.75)	26 (21.75–48)	0.265
Heart rate (bpm)		94.0 (73.25–100.0)	86.5 (66.25–106.5)	0.956
Temperature (°C)		36.55 (36.08–37.65)	36.95 (36.40–37.48)	0.515
SpO2/FiO2		117 (114–148)	88 (98–120)	0.025
**Biology**				
Hemoglobin (g/dL)		12.7 (11.4–13.7)	12.2 (11.3–13.7)	0.832
Leucocyte (/mm^3^)		7.4 (5.8–12.6)	8.0 (6.0–9.0)	0.061
Neutrophils (/mm^3^)		5.4 (4.4–10.4)	6.0 (4.6–9.1)	0.944
Lymphocyte (/mm^3^)		0.71 (0.49–1.27)	0.72 (0.44–1.06)	0.152
Fibrinogen (g/L)		6.2 (4.8–6.8)	7.1 (5.2–7.9)	0.316
Creatinine (µmol/L)		108.5 (82.0–140.0)	94 (77.0–144.0)	0.276
C reactive protein (mg/L)		118.0 (70.9–153.5)	108.5 (64.4–190.5)	0.813
Procalcitonin (µg/L)		0.37 (0.16–8.66)	0.59 (0.21–1.51)	0.053
Lactate (g/L)		2.2 (1.3–3.1)	1.9 (1.4–2.1)	0.302
Albumin (mg/L)		27 (23–33)	24 (22–27)	0.049
**Acute management**				
Corticosteroids		15 (60)	37 (90)	0.009
Antibiotics		23 (92)	38 (93)	1.000

HCM, high-concentration mask; HFNCO, high-flow nasal cannula oxygenation; CAP, community-acquired pneumonia; NHAP, nursing-home-acquired pneumonia; WHO severity index, world health organization severity index [19]; ARF, acute respiratory failure; NEWS2, National Early Warning score [18]; SD, standard deviation.

**Table 2:** Association between high-flow nasal cannula oxygenation (HFNCO) and in-hospital outcome in SARS-CoV-2-related acute respiratory failure, before and after adjustment for confounders by stabilized inverse probability weighting.

	Crude Hazard Ratio		Weighted Hazard Ratio	
Variable	HR (95% CI)	*p*	HR (95% CI)	*p*
Survival at 30 days	0.58 (0.34–0.99)	0.04	0.57 (0.33–0.99)	0.04
Morphine introduction	0.40 (0.23–0.70)	0.002	0.39 (0.21–0.71)	0.002
Midazolam introduction	0.71 (0.40–1.28)	0.26	0.66 (0.37–1.19)	0.17

## Data Availability

The data presented in this study are available on request from the corresponding author. The data are not publicly available.
